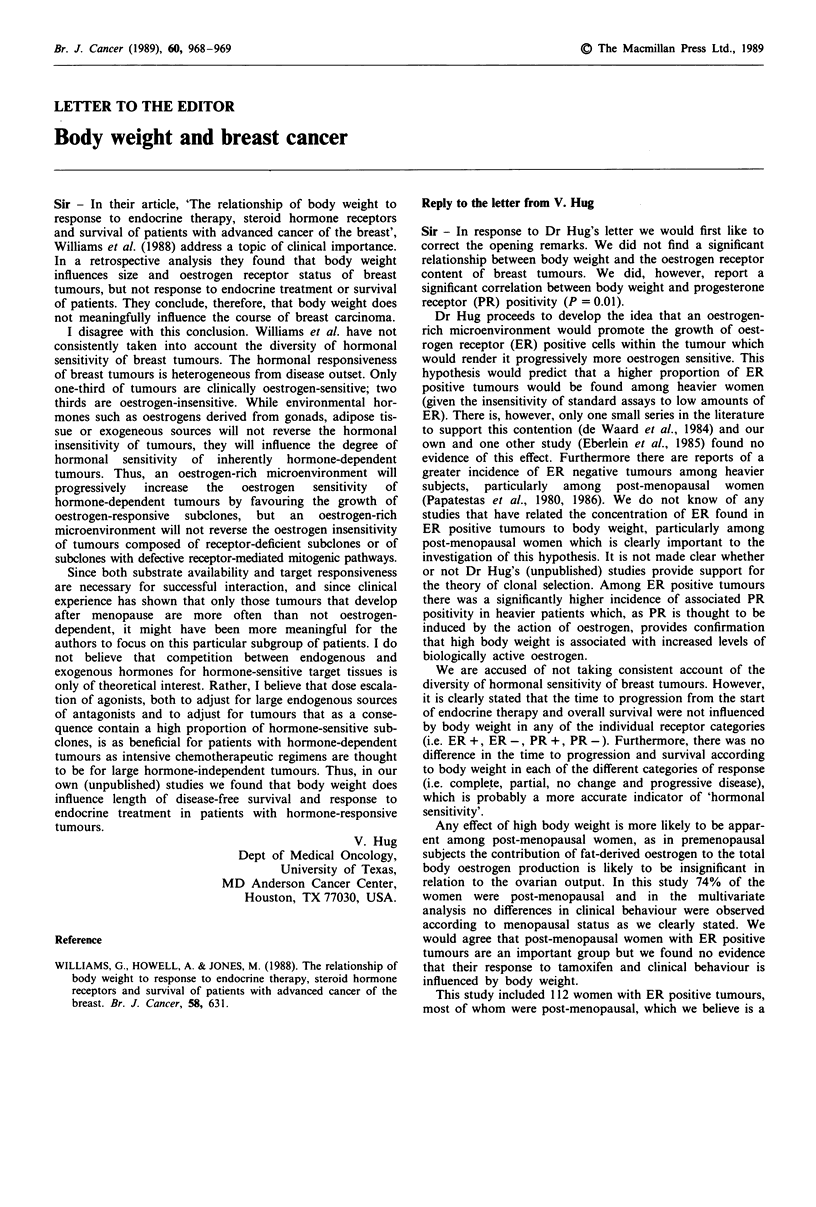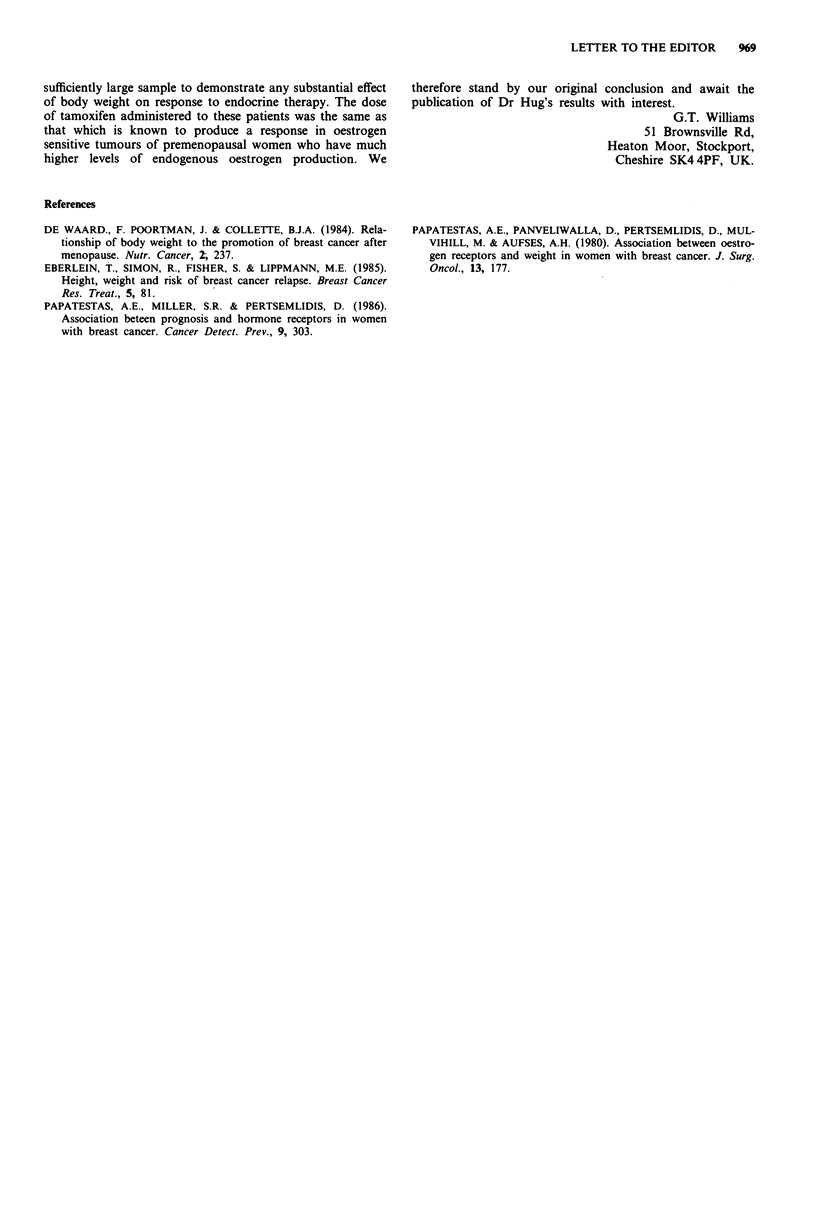# Reply to the letter from V. Hug

**Published:** 1989-12

**Authors:** G.T. Williams


### Reference

WILLIAMS, G., HOWELL, A. & JONES, M. (1988). The relationship of body weight to response to endocrine therapy, steroid hormone receptors and survival of patients with advanced cancer of the breast. *Br. J. Cancer*, **58**, 631.

### Reply to the letter from V. Hug

**Sir** – In response to Dr Hug's letter we would first like to correct the opening remarks. We did not find a significant relationship between body weight and the oestrogen receptor content of breast tumours. We did, however, report a significant correlation between body weight and progesterone receptor (PR) positivity ($P = 0.01$).

Dr Hug proceeds to develop the idea that an oestrogen-rich microenvironment would promote the growth of oestrogen receptor (ER) positive cells within the tumour which would render it progressively more oestrogen sensitive. This hypothesis would predict that a higher proportion of ER positive tumours would be found among heavier women (given the insensitivity of standard assays to low amounts of ER). There is, however, only one small series in the literature to support this contention (de Waard *et al.*, 1984) and our own and one other study (Eberlein *et al.*, 1985) found no evidence of this effect. Furthermore there are reports of a greater incidence of ER negative tumours among heavier subjects, particularly among post-menopausal women (Papatestas *et al.*, 1980, 1986). We do not know of any studies that have related the concentration of ER found in ER positive tumours to body weight, particularly among post-menopausal women which is clearly important to the investigation of this hypothesis. It is not made clear whether or not Dr Hug's (unpublished) studies provide support for the theory of clonal selection. Among ER positive tumours there was a significantly higher incidence of associated PR positivity in heavier patients which, as PR is thought to be induced by the action of oestrogen, provides confirmation that high body weight is associated with increased levels of biologically active oestrogen.

We are accused of not taking consistent account of the diversity of hormonal sensitivity of breast tumours. However, it is clearly stated that the time to progression from the start of endocrine therapy and overall survival were not influenced by body weight in any of the individual receptor categories (i.e. ER +, ER −, PR +, PR −). Furthermore, there was no difference in the time to progression and survival according to body weight in each of the different categories of response (i.e. complete, partial, no change and progressive disease), which is probably a more accurate indicator of 'hormonal sensitivity'.

Any effect of high body weight is more likely to be apparent among post-menopausal women, as in premenopausal subjects the contribution of fat-derived oestrogen to the total body oestrogen production is likely to be insignificant in relation to the ovarian output. In this study 74% of the women were post-menopausal and in the multivariate analysis no differences in clinical behaviour were observed according to menopausal status as we clearly stated. We would agree that post-menopausal women with ER positive tumours are an important group but we found no evidence that their response to tamoxifen and clinical behaviour is influenced by body weight.

This study included 112 women with ER positive tumours, most of whom were post-menopausal, which we believe is a sufficiently large sample to demonstrate any substantial effect of body weight on response to endocrine therapy. The dose of tamoxifen administered to these patients was the same as that which is known to produce a response in oestrogen sensitive tumours of premenopausal women who have much higher levels of endogenous oestrogen production. We therefore stand by our original conclusion and await the publication of Dr Hug's results with interest.

G.T. Williams
51 Brownsville Rd,
Heaton Moor, Stockport,
Cheshire SK4 4PF, UK.

**References**

DE WAARD., F. POORTMAN, J. & COLLETTE, B.J.A. (1984). Relationship of body weight to the promotion of breast cancer after menopause. *Nutr. Cancer*, **2**, 237.

EBERLEIN, T., SIMON, R., FISHER, S. & LIPPMANN, M.E. (1985). Height, weight and risk of breast cancer relapse. *Breast Cancer Res. Treat.*, **5**, 81.

PAPATESTAS, A.E., MILLER, S.R. & PERTSEMLIDIS, D. (1986). Association beteen prognosis and hormone receptors in women with breast cancer. *Cancer Detect. Prev.*, **9**, 303.

PAPATESTAS, A.E., PANVELIWALLA, D., PERTSEMLIDIS, D., MULVIHILL, M. & AUFSES, A.H. (1980). Association between oestrogen receptors and weight in women with breast cancer. *J. Surg. Oncol.*, **13**, 177.